# Introducing an alternative nonlinear model to characterize the growth curve in ostrich

**DOI:** 10.1016/j.psj.2024.104465

**Published:** 2024-10-31

**Authors:** Navid Ghavi Hossein-Zadeh

**Affiliations:** Department of Animal Science, Faculty of Agricultural Sciences, University of Guilan, Rasht 41635-1314, Iran

**Keywords:** Goodness-of-fit, Growth function, Growth rate, Model fitting, Ostrich

## Abstract

By applying a sinusoidal function (as a trigonometric model), this study aimed to introduce this function into ostrich weight development research, using ostrich growth data from the literature and comparing it with some routinely used growth models such as monomolecular, Bridges, Janoschek, logistic, Von Bertalanffy, Richards, Schumacher, Morgan, Chanter, and Weibull. During the fitting of nonlinear regression curves, model performance was evaluated and model behavior was examined. Body weight data of the domestic ostriches used in this study were reported in the Blue Mountain Ostrich Nutrition e-bulletin from three different studies (data sets 1 to 3). In all data sets, body weight was measured monthly from one to twelve months of age. The adjusted coefficient of determination, root mean square error, Akaike's information criterion, and Bayesian information criterion were used to evaluate each model's overall goodness-of-fit to different data profiles. Based on the goodness-of-fit criteria, the sinusoidal model was determined to be the most suitable function for fitting the growth curve of ostriches in data sets 1 and 2. However, both monomolecular and logistic models had the worst fit to the growth curve of ostriches in these data sets. For data set 3, the Weibull model provided the best fit of the growth curve of ostriches, but the sinusoidal function had the worst fit. Absolute growth rate (AGR), calculated using the first derivative of the best model with time showed that AGR values ​​increased with age until days 174, 90, and 68 for data sets 1 to 3, respectively, and then decreased. Overall, this study offers implications for advancing research on ostrich production systems and providing insightful information on the application of alternative nonlinear models in modeling ostrich growth.

## Introduction

The ostrich (*Struthio camelus*) has gained prominence in the livestock sector, symbolizing a unique blend of agricultural output, eco-friendly management, and financial prospects. As an alternative source of protein that supports human health, ostrich farming is a growing sector of the poultry industry (Al-Khalifa and Al-Naser, 2014; Abbas et al., 2017; Brassó et al., 2021). However, some of the future food needs of developing countries can be met by paying attention to the ostrich farming industry in these countries (Abbas et al., 2018a,b,c). Ostrich meat is a lean, nutrient-rich substitute that has become more and more popular as consumer preferences are shifting toward healthier protein sources (Miah et al., 2020). Simultaneously, farmers benefit from an extra source of income due to the demand for ostrich leather, which is prized for its softness, toughness, and exotic appeal—particularly in the fashion accessory industry. Ostriches contribute to food security, job creation, and rural economic development in a variety of regions, especially when combined with the value of their feathers for ornamentation and decorative purposes. To achieve efficient growth, it is essential to understand the ostrich's ability to meet specific nutritional needs when used as a livestock animal (Cooper, 2005). Compared to poultry, ostriches have a higher feed conversion rate, which affects growth (Cilliers, 1995).

Efficient management strategies are essential for success in this cutthroat market. A thorough understanding of the growth patterns that ostriches exhibit is essential to these practices, as these patterns are influenced by a wide range of factors, including age, sex, genetic predisposition, nutritional management, and environmental conditions (Bovera et al., 2011; Mahrose et al., 2015). Ostrich growth performance is fundamental to productivity and profitability of ostrich farms, particularly in the crucial early growth stages. Understanding the different biological and environmental factors that affect the health and productivity of the birds is essential for effective management in ostrich farming. Growth is a crucial factor in this context, as it directly influences the timeliness and caliber of products that are ready for the market. Therefore, it is crucial to create accurate growth curve models to forecast the growth patterns of birds throughout their life cycle (Ghavi Hossein-Zadeh and Golshani, 2016). Farm managers can optimize performance and resource utilization by using these models to make informed decisions about breeding programs, housing conditions, and feeding schedules (da Silva Marinho et al., 2013). Additionally, knowing the ostrich growth curves can have management implications that go beyond individual farms. Growth modeling insights can influence industry-wide procedures and help establish benchmarks for product quality, reproductive efficiency, and animal welfare. Robust growth analysis will be essential to ensuring sustainable practices and optimizing economic returns for producers as the ostrich industry works to meet growing global demand.

Conventional growth models are frequently employed in research on livestock growth, but they might not fully represent the intricacies and intrinsic variability of animal growth patterns. This shortcoming emphasizes the necessity for substitute nonlinear models that can more faithfully capture the biological realities of growth (Ghavi Hossein-Zadeh, 2024). Ostrich growth trajectories are unique, and nonlinear growth models can account for them by considering variables like age, sex, dietary intake, and environmental conditions. Through the use of these sophisticated modeling techniques, scientists can improve breeding strategies that boost growth rates and overall productivity by deepening our understanding of growth dynamics. Researchers can also more effectively account for individual variability in growth performance by using alternative nonlinear growth models. This is important because it allows management strategies to be customized to meet unique breeding goals and environmental constraints (Ghavi Hossein-Zadeh, 2024). The suitability of a sinusoidal function (as an alternative) to model the growth curve of broiler chickens, dairy heifers, ducks, and turkeys has been documented in earlier studies (Darmani Kuhi et al., 2018, 2019a,b,c; Ghavi Hossein-Zadeh, 2024). Furthermore, the growth curves of different ostrich populations have only been reported in a limited number of studies, and most previous studies employed conventional nonlinear models. Consequently, there is a dearth of research on ostrich growth curves in the literature, especially when other models like sinusoidal models are applied. By applying the sinusoidal function to ostrich growth data from the literature and comparing it with 10 standard growth functions, viz. monomolecular, Bridges, Janoschek, logistic, Morgan, von Bertalanffy, Richards, Schumacher, Chanter, and Weibull, the current study was the first to employ a sinusoidal function in ostrich weight development studies.

## Materials and methods

### Data sources

Body weight records of the domestic ostriches used in this study were reported in the Blue Mountain Ostrich Nutrition e-bulletin No. 81 (Benson, 2002) from three different data sets by Benson (2002); Cilliers (1995), and Degen et al. (1991). In all studies, body weight was measured monthly from one to twelve months of age (Supplementary Table 1).

### Nonlinear models

The following models were fitted to the data to model the relationship between body weight and age of ostriches: monomolecular, Bridges, Janoschek, logistic, Morgan, von Bertalanffy, Richards, Schumacher, Chanter, Weibull, and sinusoidal (Table 1).

**Table 1:** Functional forms of nonlinear models for describing the growth curve of ostriches

Model	Functional form
Monomolecular	$y=\frac{a}{1+e^{\left(b-kt\right)}}$
Bridges	$y=W_{0}+\left(1-e^{-kt^{m}}\right)$
Janoschek	$y=a-(\left(a-W_{0}\right)e^{-kt^{m}})$
Von Bertalanffy	$y=a{\left(1-be^{-kt}\right)}^{3}$
Richards	$y=\frac{a}{{\left(1-be^{-kt}\right)}^{\frac{1}{m}}}$
Schumacher	$y=\frac{ab^{2}k}{{\left(t+b\right)}^{2}}e^{\left(\frac{bkt}{t+b}\right)}$
Morgan	$y=ab^{k}k\frac{t^{k-1}}{{\left(t^{k}+b^{k}\right)}^{2}}$
Logistic	$y=\frac{a}{1+be^{-kt}}$
Chanter	$y=\frac{W_{0}b}{W_{0}+\left(b-W_{0}\right)e^{\left(\frac{-m}{d}\right)\left(1-e^{-dt}\right)}}$
Weibull	$y=a-\left(a-b\right)e^{-\left(\frac{k-1}{k}\right){\left(\frac{t}{IP}\right)}^{k}}$
Sinusuidal	$y=y_{0}+a\times \sin\left(\frac{2\pi t}{b}+c\right)$

y= represents body weight at age t (day); a= represents asymptotic weight, which is interpreted as mature weight; and b= is an integration constant related to initial animal weight. The value of b is defined by the initial values for y and t; k= is the maturation rate, which is interpreted as weight change in relation to mature weight to indicate how fast the animal approaches adult weight; m= is the parameter that gives shape to the curve by indicating where the inflection point occurs; d= is the constant parameter; IP= is inflection point; W_0_= is initial body weight. For sinusoidal function, *a* is the amplitude, $y_{o}$ is the vertical offset and *c* is the phase shift. This sinusoidal function is periodic with period *b*. Also, for sinusoidal function, W_0_=y_0_+a × sin(c), and final weight is calculated by a+ y_0_. For the Schumacher and Morgan models, the asymptotic weight is not an estimated parameter.

### Statistical analysis

The parameters of nonlinear models were estimated through independent fitting of the models to ostrich body weight records using the NLIN and MODEL procedures in SAS (SAS Institute, 2002). Iteration techniques for fitting non-linear functions were based on the Gauss-Newton method. To facilitate the iterative process, specific initial values for the parameters needed to be defined. The final estimates were unaffected by the initial values that were chosen.

The quality of predictions and goodness-of-fit for each model were assessed using the adjusted coefficient of determination ($R_{adj}^{2}$), residual standard deviation or root mean square error (RMSE), Durbin-Watson statistic (DW), Akaike's information criterion (AIC), and Bayesian information criterion (BIC).

The formula that was utilized for the calculation of $R_{adj}^{2}$was:

$$R_{adj}^{2}=1-\left[\frac{\left(n-1\right)}{\left(n-p\right)}\right]\left(1-R^{2}\right)$$

Where the multiple coefficient of determination is represented by $R^{2}$($R^{2}=1-\frac{RSS}{TSS}$), n represents the number of observations (data points), p represents the number of parameters, RSS denotes the residual sum of squares, and TSS denotes the total sum of squares. The $R^{2}$value is utilized to calculate the proportion of variability in the trait's total variation that can be explained by the growth curve model. The $R^{2}$ value is always between 0 and 1, with the model considered satisfactory when $R^{2}$ approaches 1.

RMSE is calculated through the following formula:

$$RMSE=\frac{RSS}{\sqrt{n-p-1}}$$

The RSS is the sum of squared errors in the data. A model with the lowest RMSE is considered the most favorable.

To assess the normality of the residuals of the fitted nonlinear models, the Shapiro-Wilk test was used. This test is commonly used to examine whether the residuals follow a normal distribution. A significant P-value (P<0.05) indicates deviations from normality. To check the assumption of constant variance in the residuals, the White's test was also used. White's test is a common statistical test for heteroscedasticity that evaluates whether the variance of the residuals remains stable across different levels of the independent variable. A non-significant result of the White's test (P>0.05) would indicate that the homoscedasticity assumption is met, supporting the validity of the regression models.

Autocorrelation of the residuals from the regression analysis was evaluated using the Durbin-Watson (DW) statistic. If autocorrelated residuals are present, it suggests that the model may not be appropriate for the data. The DW statistic ranges from 0 to 4, with a value near two indicating no autocorrelation, a value near 0 indicating positive autocorrelation, and a value near 4 indicating negative autocorrelation (Ghavi Hossein-Zadeh, 2014). DW was determined using the following formula:

$$DW=\frac{\sum_{t}^{n}{\left(e_{t}-e_{t-1}\right)}^{2}}{\sum_{t=1}^{n}e_{t}^{2}}$$

Where $e_{t}$represents the residual at time e, and $e_{t-1}$ represents the residual at time t-1.

The AIC was calculated using the following formula (Burnham and Anderson, 2002):

AIC=n×ln(RSS)+2p

AIC is a valuable statistic for comparing models with different levels of complexity. A lower AIC value means a better fit when comparing models.

BIC combines maximum likelihood and model selection by including a penalty for the model complexity in addition to the likelihood function:

$$BIC=n\ln\left(\frac{RSS}{n}\right)+p\ln\left(n\right)$$

A better fit is indicated by a lower BIC value when comparing models.

Once the optimal function was chosen, the absolute growth rate, or AGR, was calculated using the function's first derivative concerning time ($\frac{\partial y}{\partial t}$). AGR is a useful tool for calculating the average growth rate of a population. In this case, this indicates the approximate daily weight gain during a growth phase (Ghavi Hossein-Zadeh, 2015). In addition, the inflection point (IP) was calculated using the second derivative of the best function concerning time (Mischan et al., 2011; Teixeira et al., 2021). As stated by Mischan et al. (2011), IP is the value at which the growth model's second derivative is set to zero. The first and second derivatives of the best function were calculated using R software version 4.4.0.

## Results

Table 2 displays the estimated parameters of the nonlinear growth models for ostriches. Goodness-of-fit statistics for the eleven growth models fitted to body weight records of ostriches are also presented in Table 3. Although there were minimal differences in $R_{adj}^{2}$ values between the models, the sinusoidal model had the highest $R_{adj}^{2}$ value for data sets 1 and 2. However, both monomolecular and logistic models produced the smallest values of $R_{adj}^{2}$ for these data sets (Table 3). On the other hand, for data set 3, other models except the sinusoidal function, which gave a negative $R_{adj}^{2}$ value, had minimal differences in $R_{adj}^{2}$, and the Weibull model had the highest value (Table 3). DW values ranged from 0.6640 (for logistic) to 2.0012 (for sinusoidal) in data set 1, from 0.8715 (for logistic) to 2.0976 (for sinusoidal) in data set 2, and from 0.2117 (for sinusoidal) to 1.5884 (for Bridges) in data set 3. For data set 1, the Bridges, Janoschek, Richards, Chanter, Weibull, and sinusoidal models had no autocorrelation between residuals. For data set 2, only the Morgan and sinusoidal models had no autocorrelation between residuals. On the other hand, for data set 3, the Bridges, Janoschek, and Richards models presented no autocorrelation between residuals (Table 3). DW values for other models indicated positive autocorrelation between residuals. For data set 1, except the monomolecular and logistic models, other functions had a normal residual distribution (P>0.05). However, all models produced a normal residual distribution for data sets 2 and 3 (P>0.05). The results of White's test examining the assumption of constant variance in the residuals confirmed residual homogeneity for all models in data sets 1 to 3 (P>0.05) (Table 3). For data sets 1 and 2, the monomolecular and logistic models had the highest RMSE, AIC, and BIC values, while the sinusoidal function produced the lowest RMSE, AIC, and BIC values for both data sets (Table 3). As a result, the sinusoidal model was determined to be the most suitable function for fitting the growth curve of ostriches for data sets 1 and 2. However, both monomolecular and logistic models had the worst fit to the growth curve of ostriches for these data sets. For data set 3, the Weibull model produced the lowest RMSE, AIC, and BIC values, and consequently provided the best fit of the growth curve of ostriches, but the sinusoidal function had the highest RMSE, AIC, and BIC values, and provided the worst fit.

**Table 2:** Parameter estimates for the different growth models in ostriches

Data set	Parameter	Model										
		Monomolecular	Bridges	Janoschek	Logistic	Von Bertalanffy	Richards	Schumacher	Morgan	Chanter	Weibull	Sinusuidal
Data set 1*	y_0_	-	-	-	-	-	-	-	-	-	-	49.3647
	W_0_	-	2.8732	2.8732	-	-	-	-	-	1.9078	-	-
	a	104.5	108.1	110.9	104.5	131.0	116.5	14.9768	-78925.9	-	110.9	51.3537
	b	2.9920	-	-	19.9255	0.8580	-0.3656	139.8	574.0	-80.3769	2.8732	843.2
	c	-	-	-	-	-	-	-	-	-	-	4.9835
	d	-	-	-	-	-	-	-	-	0.0116	-	-
	k	0.0163	0.00002	0.00002	0.0163	0.00656	0.00964	0.0706	-2.5693	-	1.9516	-
	m	-	1.9516	1.9516	-	-	0.0776	-	-	-	-	-
	μ	-	-	-	-	-	-	-	-	0.0375	-	-
	IP	-	-	-	-	-	-	-	-	-	161.0	-
Data set 2⁎⁎	y_0_	-	-	-	-	-	-	-	-	-	-	27.3143
	W_0_	-	-1.3213	-1.3213	-	-	-	-	-	2.9416	-	-
	a	101.6	114.2	112.9	101.6	115.5	119.9	31.0810	-84449.2	-	112.9	73.0858
	b	2.6014	-	-	13.4828	0.7968	0.9730	134.8	589.9	1412.2	-1.3213	1178.1
	c	-	-	-	-	-	-	-	-	-	-	5.8046
	d	-	-	-	-	-	-	-	-	0.00999	-	-
	k	0.0167	0.00035	0.00035	0.0167	0.00802	0.00692	0.0661	-2.3239	-	1.4904	-
	m	-	1.4904	1.4905	-	-	-0.4954	-	-	-	-	-
	μ	-	-	-	-	-	-	-	-	0.0370	-	-
	IP	-	-	-	-	-	-	-	-	-	98.9849	-
Data set 3⁎⁎⁎	y_0_	-	-	-	-	-	-	-	-	-	-	81.2345
	W_0_	-	-16.8017	-16.8016	-	-	-	-	-	4.8485	-	-
	a	140.6	250.2	233.4	140.6	162.3	216.4	66.7655	-167448	-	-0.9938	14.7996
	b	2.4877	-	-	12.0333	0.7723	1.0779	153.6	759.6	334.0	467.6	-45.0773
	c	-	-	-	-	-	-	-	-	-	-	-67.0616
	d	-	-	-	-	-	-	-	-	0.00785	-	-
	k	0.0154	0.00284	0.00284	0.0154	0.00739	0.00324	0.0559	-2.1897	-	-0.5283	-
	m	-	0.9948	0.9948	-	-	-0.9405	-	-	-	-	-
	μ	-	-	-	-	-	-	-	-	0.0321	-	-
	IP	-	-	-	-	-	-	-	-	-	68.2845	-

⁎ From the study of Cilliers (1995);

⁎⁎ From the study of Degen et al. (1991);

⁎⁎⁎ From the study of Benson (2002)

**Table 3:** Comparing goodness-of-fit for different growth curves in ostriches

Data set	Statistics	Model										
		Monomolecular	Bridges	Janoschek	Logistic	Von Bertalanffy	Richards	Schumacher	Morgan	Chanter	Weibull	Sinusoidal
Data set 1*	$R_{adj}^{2}$	0.9974	0.9995	0.9995	0.9974	0.9987	0.9995	0.9993	0.9997	0.9996	0.9995	0.9998
	DW	0.6642	1.7864	1.7864	0.6640	1.0101	1.6731	1.3309	1.3687	1.7562	1.7864	2.0012
	Shapiro-Wilk (P-value)	0.0331	0.3792	0.3795	0.0329	0.2579	0.8534	0.6896	0.6690	0.6852	0.3786	0.3102
	White's test (P-value)	0.1633	0.6002	0.6256	0.1633	0.2883	0.3488	0.6435	0.2971	0.3236	0.4417	0.3092
	RMSE	1.7628	0.7461	0.7461	1.7628	1.2149	0.8019	0.9004	0.6321	0.7044	0.7461	0.5062
	AIC	45.9725	25.9232	25.9232	45.9725	37.0386	27.6561	29.8482	21.3595	24.5443	25.9232	16.6123
	BIC	17.6083	-1.9561	-1.9561	17.6083	8.6745	-0.2232	1.4841	-7.0046	-3.3350	-1.9561	-11.2629
Data set 2⁎⁎	$R_{adj}^{2}$	0.9912	0.9982	0.9982	0.9912	0.9979	0.9978	0.9976	0.9985	0.9965	0.9982	0.9991
	DW	0.8717	1.4102	1.4103	0.8715	1.4022	1.3224	1.3894	1.5448	1.2871	1.4102	2.0976
	Shapiro-Wilk (P-value)	0.1899	0.9647	0.9648	0.1890	0.8781	0.9815	0.5500	0.4799	0.6051	0.9648	0.5243
	White's test (P-value)	0.1537	0.9867	0.9855	0.1537	0.9036	0.9850	0.2435	0.2845	0.1595	0.9853	0.9502
	RMSE	3.0898	1.3897	1.3897	3.0898	1.5132	1.5450	1.6194	1.2686	1.9602	1.3897	0.9665
	AIC	59.4411	40.8511	40.8511	59.4412	42.3082	43.3937	43.9360	38.0774	49.1069	40.8511	32.1362
	BIC	31.0770	12.9718	12.9718	31.0770	13.9440	15.5144	15.5719	9.7132	21.2276	12.9718	4.2569
Data set 3⁎⁎⁎	$R_{adj}^{2}$	0.9746	0.9952	0.9952	0.9746	0.9917	0.9953	0.9892	0.9907	0.9867	0.9962	-0.2942
	DW	0.6579	1.5884	1.5880	0.6580	0.8646	1.4966	0.7998	0.8253	0.7739	1.4114	0.2117
	Shapiro-Wilk (P-value)	0.7560	0.5819	0.5816	0.7558	0.3830	0.6450	0.2054	0.4220	0.3105	0.5772	0.1142
	White's test (P-value)	0.1886	0.3522	0.2843	0.1888	0.2254	0.3337	0.5659	0.1071	0.2008	0.4233	0.1678
	RMSE	7.1523	3.0994	3.0994	7.1523	4.0961	3.0771	4.6744	4.3411	5.1729	2.7675	51.0935
	AIC	79.5852	60.1027	60.1027	79.5852	66.2074	59.9286	69.3741	67.6013	72.3973	57.3838	127.3611
	BIC	51.2210	32.2234	32.2234	51.2210	37.8432	32.0494	41.0100	39.2372	44.5181	29.5045	99.4818

⁎ From the study of Cilliers (1995);

⁎⁎ From the study of Degen et al. (1991);

⁎⁎⁎ From the study of Benson (2002) $R_{adj}^{2}$: Adjusted coefficient of determination; RMSE: Root means square error; DW: Durbin–Watson; AIC: Akaike information criteria; BIC: Bayesian Information Criteria

The different functions for the ostriches' final or asymptotic body weights had different magnitudes (Table 2). The mature weight of ostriches is the asymptotic weight estimate for the majority of functions, denoted by the growth model parameter "a". The asymptotic weights of ostriches predicted using the sinusoidal function as the best-fitting model for data sets 1 and 2 were 100.7184 and 100.4001 kg, respectively. These values ​​were generally below the asymptotic body weights predicted by other models. The asymptotic weight of ostriches predicted using the Weibull function as the best-fitting model for data set 3 was -0.9938 kg. It should be noted that negative asymptotic weight has no biological meaning in the context of growth curves.

**Fig. 1:**
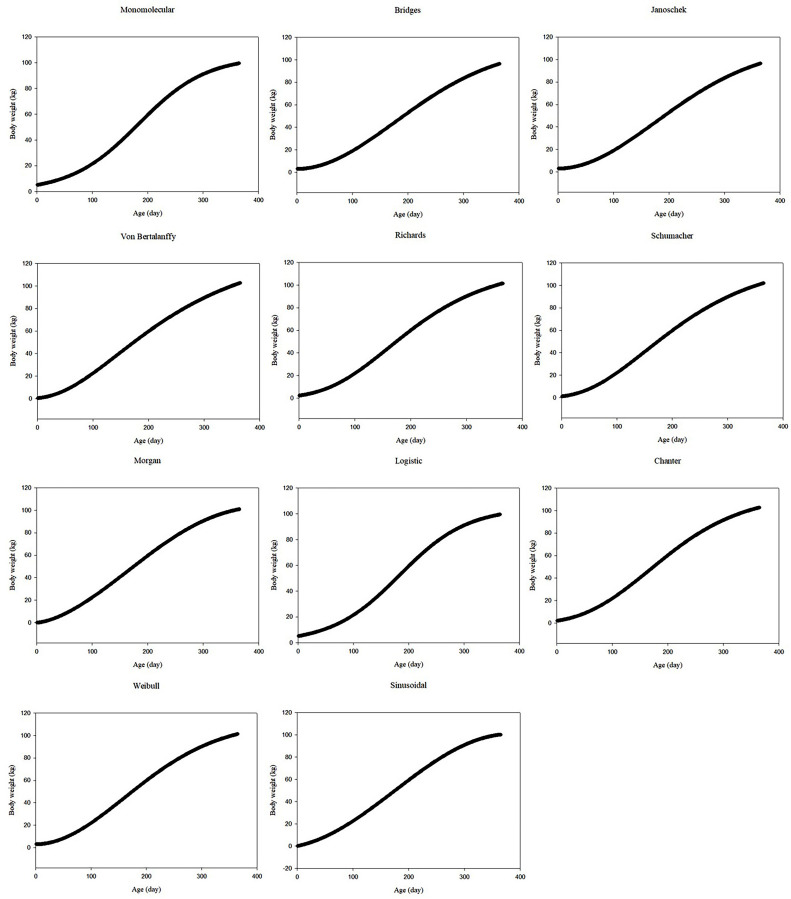
Predicted body weights of ostriches as a function of age, determined using different growth models for data set 1

**Fig. 2:**
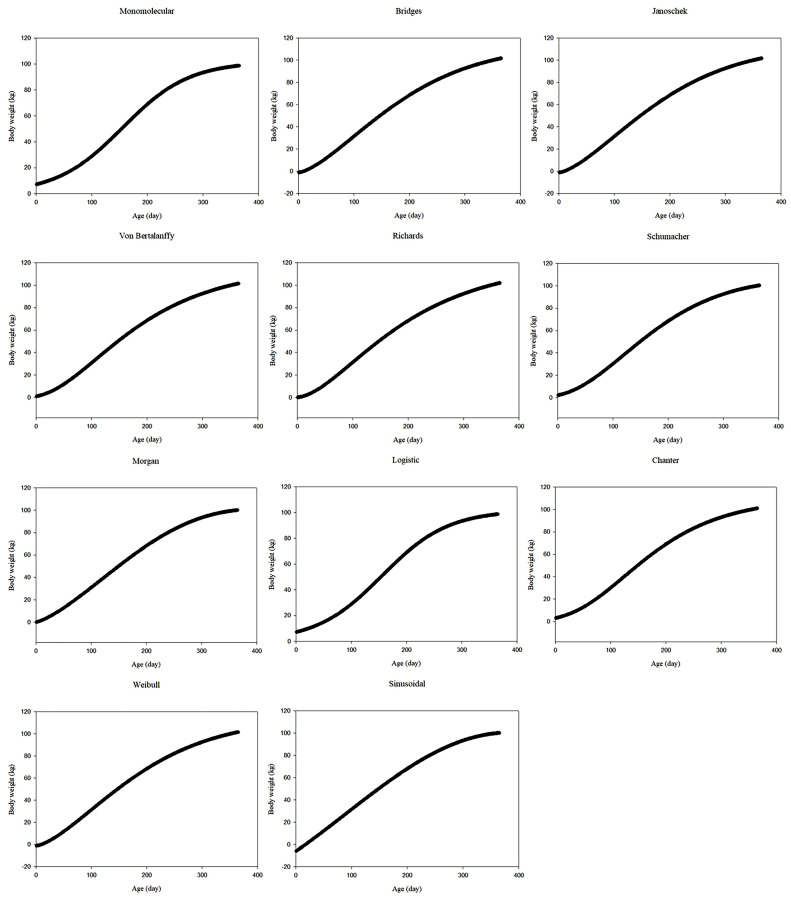
Predicted body weights of ostriches as a function of age, determined using different growth models for data set 2

**Fig. 3:**
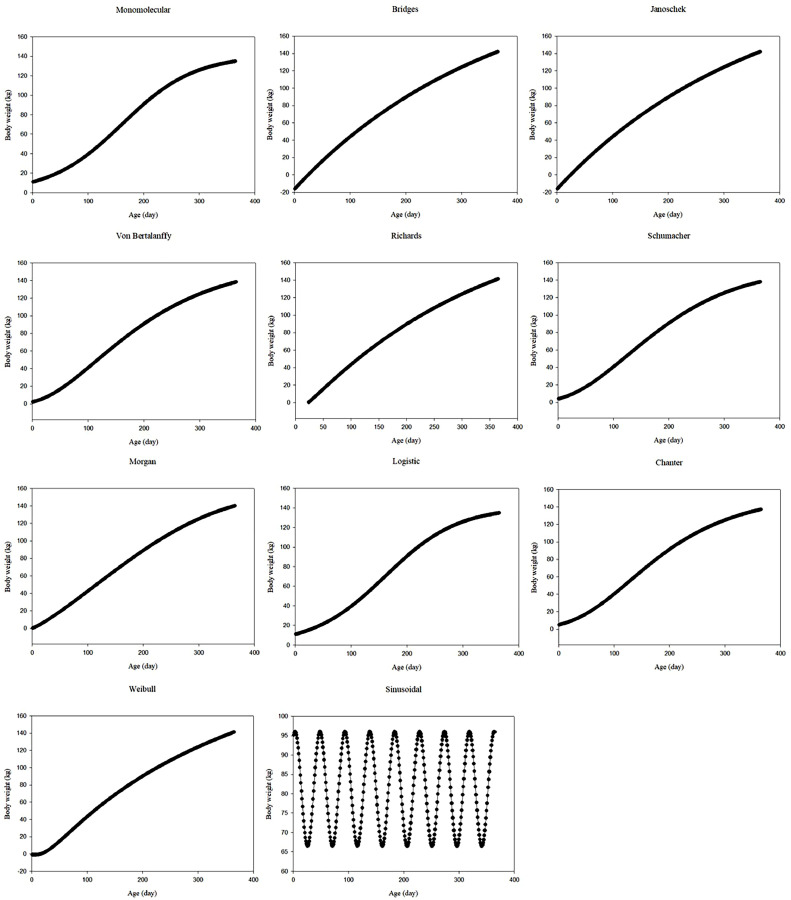
Predicted body weights of ostriches as a function of age, determined using different growth models for data set 3

The observed body weights of all data sets showed that body weight tended to increase with increasing age. Fig. 1, Fig. 2, Fig. 3 show the predicted body weights of ostriches from data sets 1 to 3 based on different growth models, respectively, as a function of age. Except for the sinusoidal function in data set 3, other functions provided generally sigmoidal increasing trends with age. For data set 1, the monomolecular, Bridges, Janoschek, Richards, logistic, Chanter, and Weibull models indicated that body weight was overestimated in the first month. The Schumacher and sinusoidal function values ​​were generally close to the first month's body weight. However, the Von Bertalanffy and Morgan models often underestimated body weight in the first month. For data set 2, the monomolecular, Richards, logistic, Von Bertalanffy, Schumacher, Morgan, and Chanter models indicated that body weight was overestimated in the first month. The Bridges, Janoschek, Weibull, and sinusoidal function values ​​were generally close to the first month's body weight. For data set 3, the monomolecular, logistic, Von Bertalanffy, Schumacher, Morgan, Chanter, and sinusoidal models often overestimated body weight in the first month. However, the Bridges, Janoschek, Richards, and Weibull models indicated that body weight was underestimated in the first month.

**Fig. 4:**
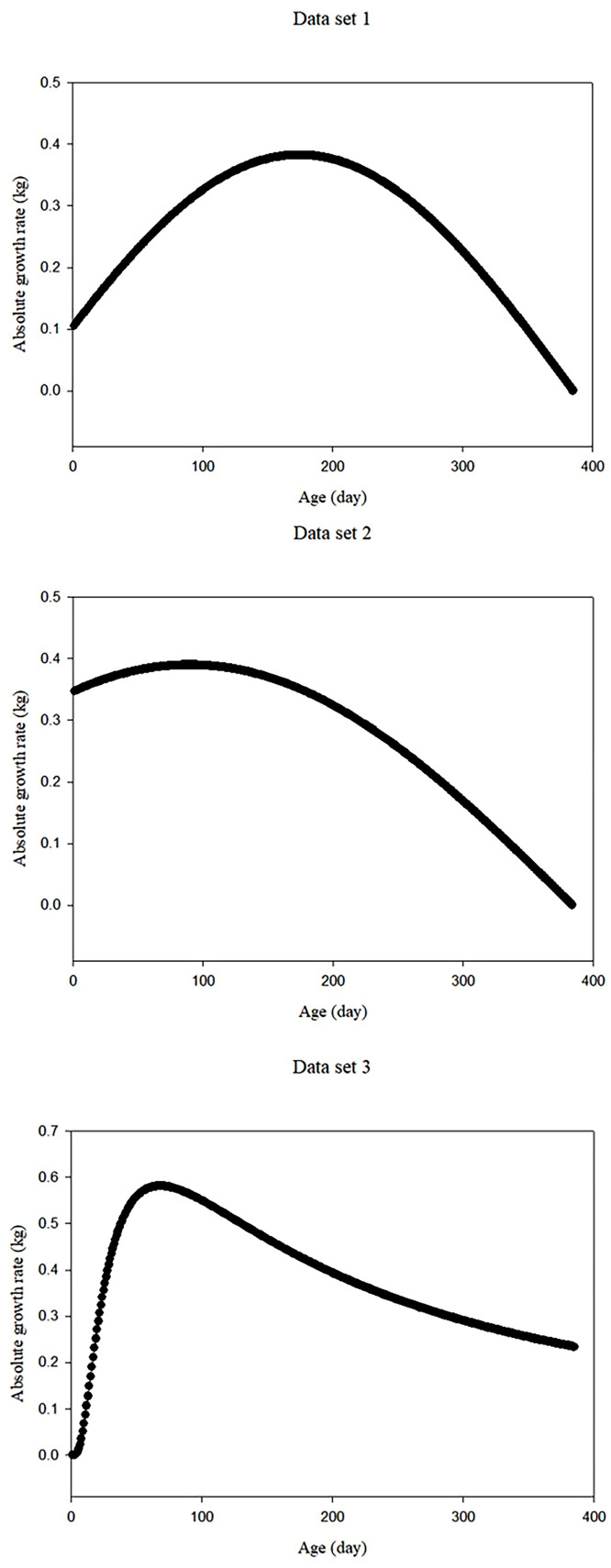
Absolute growth rate (AGR) of ostriches for data sets 1 to 3 based on the best model

Fig. 4 displays the AGR values for both data sets as a function of time, based on the first derivative of the sinusoidal model for data sets 1 and 2, and the Weibull model for data set 3. AGR values ​​increased with age until days 174, 90, and 68 for data sets 1 to 3, respectively, and then decreased. For data sets 1 to 3, based on the second derivative of the sinusoidal function, the IP was determined to be 174, 90, and 68 days old, respectively.

## Discussion

This is the first study to comprehensively model the growth curve in ostriches using classical and alternative nonlinear functions. The results of this study have important implications for ostrich and poultry farming as they open the door to better-informed and more efficient management techniques. By establishing an alternative nonlinear model as a reliable tool for precisely describing growth curves, breeders can better understand growth trajectories and thus implement optimal feeding schedules and resource allocation at the crucial developmental stages. Improved methods in selective breeding programs, where desired traits such as growth rate, feed efficiency and overall health can be prioritized based on clearly defined growth patterns, which may result from more accurate growth prediction. Additionally, the insights gained from our analysis can provide a framework for establishing growth benchmarks specific to ostrich farming as the poultry industry strives to increase productivity while ensuring animal welfare and sustainability. This would allow better breeding decisions to be made, resulting in healthier birds and more efficient production cycles. Furthermore, the use of sophisticated modeling methods such as the sinusoidal model provides opportunities to combine growth data with other physiological measures, ultimately leading to a more comprehensive understanding of ostrich development. Subsequent research could explore the use of analog models across different poultry species, which could transform growth assessment methods and lead to better outcomes in the avian sector as a whole. In the future, incorporating advanced modeling techniques could lead to better breeding results as well as more accurate and data-driven decision-making that supports the long-term growth of chicken production systems.

In this study, the sinusoidal model (as a trigonometric function) provided the best fit of the growth curve in ostriches for two data sets. Using trigonometric functions to model the growth curves of farm animals has several benefits. Livestock often exhibit oscillatory rather than strictly linear or exponential growth patterns. These periodic fluctuations in growth can be well-represented by trigonometric functions, especially sinusoidal models, which can take into account both slower developmental stages and periods of rapid growth (Darmani Kuhi et al., 2019a; Ghavi Hossein-Zadeh, 2024). These functions can be tuned to capture the distinct growth characteristics of various species or breeds by varying their amplitude, frequency, and phase. This adaptability makes it possible to develop more precise models that capture particular growth dynamics. Using trigonometric functions instead of conventional models could increase the goodness-of-fit for growth data. These models can produce more accurate predictions and insights into growth trends by offering a better fit (Ghavi Hossein-Zadeh, 2024). The growth of livestock can be influenced by cyclical factors such as dietary fluctuations and seasonal changes. These periodic effects can be incorporated into trigonometric models, allowing a more complex understanding of how external factors influence growth over time. Planning feeding schedules, maximizing breeding programs, and effectively managing resources all depend on the ability to predict future growth rates, which can only be improved with accurate growth modeling utilizing trigonometric functions. The biological mechanisms underlying animal growth stages can be better understood through the use of trigonometric functions. This knowledge can help determine key times for optimization or intervention, such as implementing nutritional changes during critical growth periods (Ghavi Hossein-Zadeh, 2024). Trigonometric functions can be applied to a wide range of farm animals due to their versatility, which allows for cross-species comparisons. In addition to promoting a deeper comprehension of livestock growth dynamics, this can also influence optimal methods for caring for animals. Trigonometric modeling can help with sustainable farming practices by optimizing feed use, reducing waste, and enhancing overall animal health and welfare. It can also provide more precise growth predictions and insights. When modeling the growth curves of farm animals, trigonometric functions can provide insightful information, but there are several limitations and restrictions to take into account. Numerous factors such as genetics, environment, nutrition, and health influence an animal's growth. Oversimplified models may be due to the inability of trigonometric functions to fully represent the interactions and complexities of these biological variables. The quality and accessibility of growth data determine the reliability of any model, including those that use trigonometric functions. Inconsistent or sparse data recording can affect the validity of the model. Because trigonometric functions are periodic in nature, they assume that growth patterns repeat regularly. However, due to various factors such as disease, changes in the environment, or management techniques, actual growth may not follow these rigid cycles, which can lead to growth patterns being misinterpreted. Differences in breed, age and habitat can cause different animals to have different growth rates. For some animals, a single trigonometric model may not be sufficient, requiring multiple models to be created for different animal groups or conditions, which would complicate analysis. Trigonometric model fitting to growth data necessitates precise parameter estimation, including amplitude, frequency, and phase. It can be difficult to accurately fit these parameters if the data does not show clear oscillatory behavior. The flexibility of trigonometric functions can lead to overfitting, a situation in which the model explains random noise in the data instead of the underlying growth trend. This may result in the model no longer being able to predict future expansions (Ghavi Hossein-Zadeh, 2024). For researchers accustomed to more conventional models, trigonometric functions may provide less intuitive results, although they work quite well for some data sets. This could affect the usefulness of the application on the farm. The time and computational effort required for fitting and simulation can increase in proportion to the complexity of the trigonometric model, especially when dealing with larger data sets. A negative $R_{adj}^{2}$for the sinusoidal model in data set 3 indicates that the model is not accurately capturing the variance in the observed growth data, suggesting that it performs worse than a simple mean prediction. This result can arise when the sinusoidal model is poorly specified for the underlying growth pattern, meaning that the periodic fluctuations assumed by the model do not match the actual growth dynamics observed in the data set. This may indicate that the data does not show a clear sinusoidal trend, possibly due to irregular growth patterns, environmental factors, or specific biological constraints affecting ostrich development that the model does not take into account. Additionally, a negative $R_{adj}^{2}$can occur if the model contains excessive parameters relative to the number of data points, leading to overfitting or indicating that the chosen model complexity is inappropriate for the data set. In this case, it serves as a warning to reconsider model selection and perhaps explore alternative models that may better fit the growth data and provide more accurate predictions.

The Weibull model, which provided the best fit for data set 3, suggests that the growth trajectory of ostriches in this particular data set may have had a gradual onset with a more consistent approximation to asymptotic weight, rather than the oscillatory dynamics present in data sets 1 and 2. The Weibull model is widely used to model growth curves, including ostriches, due to its flexibility in representing different growth patterns. This allows it to capture exponential growth in early stages, followed by a slowdown as the organism approaches its maximum size. Its ability to produce sigmoidal curves makes it particularly suitable for biological growth processes, effectively modeling initial rapid growth followed by a plateau as ostriches mature. The parameters of the Weibull distribution are easy to interpret in terms of scale and shape, providing insights into growth biology, while the model itself is simple and straightforward to implement using standard statistical software. However, the Weibull model is not without limitations; It assumes a homogeneous growth response across populations, which may not take into account genetic, health, nutritional and environmental differences between individual ostriches. Furthermore, the fixed nature of its parameters may limit its applicability in dynamic environments where growth is influenced by changing management practices and nutritional changes. Although the parameters of the model have specific interpretations, they may not always intuitively relate to biological processes, potentially complicating practical applications for growth management. There is also a risk of overfitting, particularly with small data sets. Finally, in cases where growth does not have a clear sigmoid pattern—for example, when affected by dramatic seasonal changes or stress—the Weibull model may be insufficiently descriptive, leading to inaccuracies.

Few studies have examined ostrich growth trajectories using traditional nonlinear models. Carstens et al. (2014) used three linear polynomial models and seven nonlinear growth models to examine the growth response of ostrich chicks fed diets containing three different amounts of dietary protein and amino acids. Compared to other models, they found that the third-degree linear polynomial model fits the growth curves best. du Preez et al. (1992) used a Gompertz model to calculate growth curves for three groups of ostriches: from Oudtshoorn in South Africa, the Namib Desert in Namibia, and from Zimbabwe. Cilliers et al. (1995) used the Gompertz model to analyze the growth curves of ostriches from Oudtshoorn, South Africa. Their findings indicated significant changes to the growth parameters for Oudtshoorn ostriches as reported by du Preez et al. (1992). Degen et al. (1991) described the sigmoidal growth curve of the domesticated ostrich using the Gompertz equation. Ramos et al. (2013) used both linear and nonlinear functions to fit growth curves of ostrich population in Brazil. They concluded that while all nonlinear models are better in explaining the sigmoidal nature of ostrich growth, the logistic growth model provides the best fit of hens and cockerels. The differences in the quality of fit between the functions for various ostrich populations could be explained by differences in the growth curve properties. Differences in model fit between studies may be caused by differences in sample size, body weight data, breeding locations, and mathematical structures of the model. Growth curve variations can be caused by a mix of genetic and environmental factors (Ghavi Hossein-Zadeh 2024).

According to the analysis of DW values ​​from fitting nonlinear growth curve models in the current study, there were positive autocorrelations between the residuals in several models. A positive error in one observation increases the chance of a positive error in another, a phenomenon known as positive autocorrelation or serial correlation (Ghavi Hossein-Zadeh, 2014). This could be due to numerous factors such as genetics, broader environmental influences or seasonal fluctuations that affect the growth behavior of ostriches. Positive autocorrelation in the residuals may also indicate missing variables or unmodeled time trends as potential influences on ostrich growth curves. Eliminating important variables or trends from the models can cause the residuals to exhibit systematic patterns of dependence. Model misspecification can also be indicated by positive autocorrelation in the residuals. Autocorrelation in the residuals may occur if the chosen nonlinear models do not take into account all relevant factors influencing the ostrich growth curves.

The relative importance of its growth over an animal's life is indicated by AGR values. Therefore, the AGR is calculated for each animal over a certain period and along its growth trajectory. An improved and dynamic understanding of poultry growth patterns can be gained from AGR records rather than relying solely on body weight records. Knowing this makes it easier to understand growth trajectories and patterns, including periods of rapid growth, growth plateaus, and possible growth anomalies (Boonkum et al., 2024; Ghavi Hossein-Zadeh, 2024). By calculating AGR, one can determine how fast an animal is growing at a given time. This is particularly important for research on animal development, as growth rates often differ greatly in different life stages. Early detection of fluctuations in growth performance can be facilitated by AGR values. If abnormalities in growth patterns are identified early through weight gain trend monitoring, management strategies can be adjusted or immediate intervention can be made. This may require adjusting the feeding schedule, addressing health concerns, or implementing specific growth-promoting strategies (Boonkum et al., 2024; Ghavi Hossein-Zadeh, 2024). Teixeira et al. (2021) found that the derivations of nonlinear models help explain animal growth curves more clearly. The results showed that AGR peaked at the inflection point for all data sets, which coincided with the ostriches' fastest growth rate. The declining trend is explained by the steady decline in the growth rate after the inflection point. The ostriches had not gained any weight, as evidenced by their negligible AGR at the end of the analysis period. The decline in AGR could be due to poor management. In this phase, in particular, adjustments to feed management must be made to optimize animal weight gain. IP, a measure of variation in growth rate, typically refers to the change from a fast to a slower growth phase. By finding this clue, farmers can better understand an animal's growth and the possible time it takes to reach its genetic potential. By monitoring changes in growth rate, farmers can adjust their nutrition plans to suit the specific nutritional needs of their animals at different stages of growth. This can optimize feed efficiency and minimize waste, leading to more environmentally friendly farming practices.

## Conclusion

This study successfully introduced an alternative nonlinear model—specifically the sinusoidal function—for characterizing the growth curve of ostriches, alongside traditional models such as the Weibull function. The analysis demonstrated that the sinusoidal model was the best fit for growth data in two of the three data sets, suggesting that ostrich growth patterns may exhibit periodic fluctuations influenced by biological and environmental factors. Conversely, the Weibull model provided the best fit for the third data set, indicating its flexibility in capturing more gradual and stable growth trajectories. This highlights the importance of using different modeling approaches when analyzing ostrich growth, as different data sets may reflect different growth dynamics. This highlights the need for researchers and practitioners to select appropriate models tailored to the specific characteristics of their data. The results of this study go beyond purely theoretical advances because they provide valuable practical insights for ostrich breeding. By using models that accurately reflect growth behavior, producers can better predict weight gain patterns, improving management strategies, feeding schedules, and overall production efficiency. Understanding that growth patterns can vary significantly between populations or under different conditions allows producers to adapt their management practices to optimize growth performance. Furthermore, the introduction of alternative models opens opportunities for further research to explore other potential nonlinear growth functions and ultimately contributes to a more comprehensive understanding of growth dynamics in ostriches and potentially other livestock species. Finally, the ability to accurately model growth provides important data for decision-making regarding breeding, nutrition and general management practices to increase productivity on ostrich farms.

## Funding

None.

## Declaration of competing interest

The author confirms that there are no known conflicts of interest associated with this manuscript which have influenced its outcome.

## Appendix Supplementary materials

Image, application 1
