# Supplementary material for: Introducing an alternative nonlinear model to characterize the growth curve in ostrich
Source: Poult Sci. 2024 Oct 31;103(12):104465. doi: 10.1016/j.psj.2024.104465 (PMC11585753; doi:10.1016/j.psj.2024.104465)
Supplement: Supplementary file 1 [file mmc1.docx]

Supplementary Table 1. Data sets for modeling the growth curve in ostriches

| Age (day) |  | Body weight (kg) | | |
| --- | --- | --- | --- | --- |
|  |  | Data set 1^*^ | Data set 2^**^ | Data set 3^***^ |
| 30 |  | 4.0 | 4.00 | 5.7 |
| 60 |  | 11.0 | 15.90 | 16.89 |
| 90 |  | 19.5 | 28.16 | 37.31 |
| 120 |  | 28.5 | 39.45 | 55.31 |
| 150 |  | 39.5 | 50.45 | 71.2 |
| 180 |  | 52.1 | 59.40 | 83.05 |
| 210 |  | 63.4 | 71.16 | 92.41 |
| 240 |  | 73.3 | 79.46 | 105.17 |
| 270 |  | 82.4 | 88.81 | 111.72 |
| 300 |  | 91.0 | 93.42 | 120.44 |
| 330 |  | 96.3 | 96.46 | 131.81 |
| 360 |  | 99.9 | 99.91 | 143.8 |

* From the study of Cilliers (1995); ** From the study of Degen et al. (1991); *** From the study of Benson (2002)
